# Comparison of Chest Computed Tomography Between the Two Waves of Coronavirus Disease 2019 in Belgium Using Artificial Intelligence

**DOI:** 10.7759/cureus.22203

**Published:** 2022-02-14

**Authors:** Federico De Lucia, Rahim Amer Ouali, Arnaud Devriendt, Said Sanoussi, Mieke Cannie

**Affiliations:** 1 Radiology, Université Libre de Bruxelles, University Hospital Brugmann, Brussels, BEL; 2 Radiology, Centre Hospitalier Universitaire Hussein Dey d'Alger, Alger, DZA; 3 Radiology, Universitair Ziekenhuis Brussel, Vrije Universiteit Brussel, Brussels, BEL

**Keywords:** chest ct, coronavirus disease-19 (covid-19), pandemic, artificial intelligence in radiology, chest computed tomography

## Abstract

Background

In this study, we aimed to compare two outbreaks of coronavirus disease 2019 (COVID-19) in Belgium in tomographic and biological-clinical aspects with artificial intelligence (AI).

Methodology

We performed an observational retrospective study. Adult patients who were symptomatic in the first seven days with COVID-19 infection, diagnosed by chest computed tomography (CT) and/or reverse transcription-polymerase chain reaction, were included in this study. The first wave of the pandemic lasted from March 25, 2020, to May 25, 2020, and the second wave lasted from October 7, 2020, to December 7, 2020. For each wave, two subgroups were defined depending on whether respiratory failure occurred during the course of the disease. The quantitative estimation of COVID-19 lung lesions was performed by AI, radiologists, and radiology residents. The chest CT severity score was calculated by AI.

Results

In the 202 patients included in this study, we found statistically significant differences for obesity, hypertension, and asthma. The differences were predominant in the second wave. Moreover, a mixed distribution (central and peripherical) of pulmonary lesions was noted in the second wave, but no differences were noted regarding mortality, respiratory failure, complications, and other radiological and biological elements. Chest CT severity score was among the risk factors of mortality and respiratory failure. There was a mild agreement between AI and visual evaluation of pulmonary lesion extension (K = 0.4).

Conclusions

Between March and December 2020, in our cohort, for the majority of the parameters analyzed, we did not record significant changes between the two waves. AI can reduce the experience and performance gap of radiologists and better establish a hospitalization criterion.

## Introduction

The first cases of coronavirus disease 2019 (COVID-19) were described in the city of Wuhan in December 2019 when the global pandemic began. In Belgium, the first wave occurred between March and May 2020, and the second wave occurred between October 2020 and January 2021 [1]. Although severe acute respiratory syndrome coronavirus 2 (SARS-CoV-2) mutates slowly, 12,000 mutations have been described, of which the most frequent is D614G [2]. This mutation studied in vitro increases the transmissibility of the disease [3]. These adaptive mutations make it difficult to develop effective drugs and vaccines [4]. The delta variant, first found in India and then spread to England and the rest of the world, appears to carry twice the risk of hospitalization and has demonstrated moderate resistance to available vaccines [5,6]. Another recent variant is the omicron. The second wave in Belgium was characterized by a higher peak in the number of admissions to intensive care units (ICUs), but mortality remained lower compared to the first wave [1]. The radiological semiology of COVID-19 pneumopathy has been well codified. We can distinguish typical, indeterminate, and atypical signs. Among the typical signs, we cite ground-glass opacities with peripheral distribution with or without consolidation or crazy paving. There is a correlation between radiological elements, clinical data, and the temporal evolution of the disease [7]. Additionally, a significant correlation has been noted between the extension of lung disease and patient mortality [8]. The percentage of the parenchyma affected can be established by quantitative methods using software, visual qualitative methods, or semi-quantitative methods using scores [9,10]. The objective of the study is to compare the two outbreaks of COVID-19 in Belgium in tomographic and biological-clinical aspects using artificial intelligence (AI).

## Materials and methods

In this observational retrospective study, we compared the two waves of COVID-19 at the Centre Hospitalier Universitaire (CHU) Brugmann in Brussels. The periods were established based on the epidemiological curves published by Sciensano. Accordingly, the first wave lasted from March 25, 2020, to May 25, 2020, and the second wave lasted from October 7, 2020, to December 7, 2020. The cases were obtained using the Picture Archiving and Communication System. We included adult patients symptomatic for seven days or less with COVID-19 infection diagnosed using computed tomography and/or reverse transcription-polymerase chain reaction (RT-PCR). We excluded adult patients symptomatic for more than seven days, pregnant women, and minors. Subsequently, for each wave, two subgroups were defined depending on whether respiratory failure occurred during the disease. The expected sample size was 101 patients per group.

**Figure 1:**
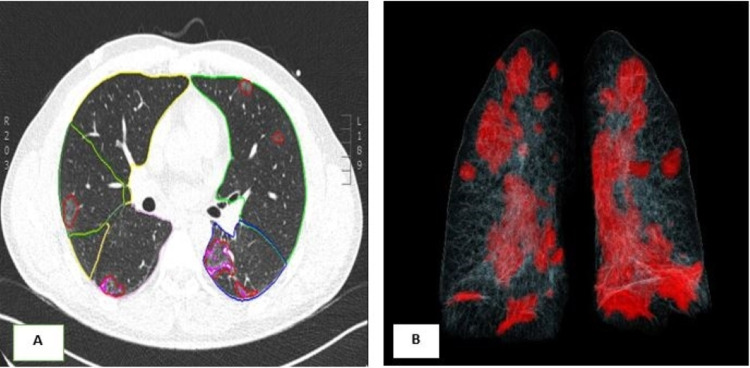
Quantitative analysis using AI. A: Ground-glass opacities tracked by AI. B: Volume-rendering reconstruction to show lung involvement. AI: artificial intelligence

For the first wave, the sampling was done randomly among 814 chest CTs without contrast injection and 100 with contrast injection. For the second wave, 711 chest CTs without contrast injection and 100 with contrast product injection were included. The clinical biological data were recorded from the patient records of CHU Brugmann. Qualitative and quantitative lung parenchyma damage on chest CT was established based on examination reports and by the experimenters. The quantitative analysis was performed using the application Pneumonia Analysis of the software Syngo.Via (Siemens Healthcare, Erlangen, Germany) with automatic contouring of the opacities based on a threshold value of density in Hounsfield unit (HU) (Figure 1). The chest CT severity score [10] was used and calculated by AI (Table 1).

**Table 1:** Chest CT severity score. CT: computed tomography

Lung lobe impairment	Points for lobe	Total severity score	Disease severity
0%	0	<7	Mild
1–5%	1	8–16	Intermediary
6–25%	2	17–25	Severe
26–50%	3		
51–75%	4		
>75%	5		

**Figure 2:**
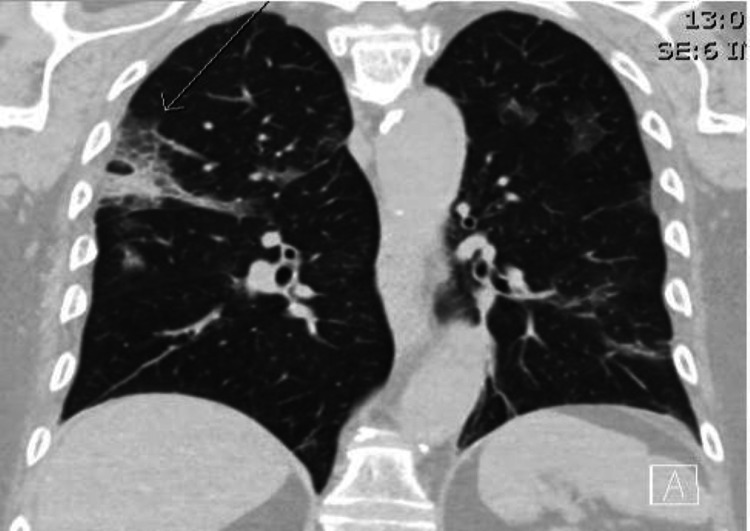
Spider web sign. CT coronal view showing ground-glass opacities, consolidation, and spider web sign (arrow). CT: computed tomography

Regarding the scanography elements, the elements considered included the presence or absence of ground-glass opacities, the distribution (central, peripheral, mixed), consolidations, crazy paving, spider web sign (Figure 2), pleural effusions, and adenopathy. We compared the pulmonary damage determined by AI to the percentage of damage visually determined by a radiologist or radiology resident. A low-dose acquisition protocol was performed using Somaton definition AS/AS+ and Somaton Drive (Siemens Healthcare, Erlangen, Germany).

The biological data were recorded from the first available blood sample after the diagnosis of COVID-19 but not after the seventh day from the onset of symptoms.

Univariate analysis was performed by comparing the two groups and subgroups intra and interwave using the t-test for continuous quantitative variables, Fisher’s exact test, or the chi-square test for discrete variables. Test k was performed for intermethod agreement on the evaluation of pulmonary damage by AI and visually. Binary logistic regression was performed for groups and subgroups with mortality and respiratory failure as independent variables and the different clinical and biological elements collected during the study as predictive variables.

## Results

In this study, 202 patients were included, 101 for each wave. The subgroups consisted of 17 patients with respiratory failure in the first wave and 14 in the second. Mortality was 23% and 27%, respectively, for the first and second waves. Among patients with respiratory failure, mortality increased to 59% for the first and 78.5% for the second wave. In the first wave, the average percentage of pulmonary damage estimated by the observer was 26% (SD = 19) and by AI was 19.7% (SD = 20); the average chest CT severity score was 10 (SD = 5). In the second wave, the corresponding values were 34.5% (SD = 22), 23.3% (SD = 21), and 11/25 (SD = 5), respectively. The percentage of hospitalization in the COVID Unit and ICU was 77% and 13% for the first wave and 78% and 20.6% for the second wave, respectively.

The intermodality concordance (observer-AI) was low (K = 0.4). Five patients required invasive ventilation in the first wave and four in the second. Between the first and second waves, the parameters that significantly differed included high blood pressure (p = 0.046), obesity (p = 0.038), and asthma (p = 0.09), which were predominant in the second wave. Among the subgroups of the first wave, significant differences were seen in crazy paving (p = 0.001), consolidation (p = 0.002), complications (p = 0.0001), ICU stay (p = 0.0001), mortality (p = 0.001), high blood pressure (p = 0.029), Singo.Via percentage (p = 0.0001), number of affected lobes (p = 0.04), and the chest CT severity score (p = 0.0001). Among the subgroups of the second wave, significant differences were noted in pleural effusion (p = 0.044), complications (p = 0.0001), hospitalization (p = 0.032), ICU stay (p = 0.028), mortality (p = 0.0001), heart problems (p = 0.001), diabetes (p = 0.0001), hypertension (p = 0.015), neoplastic history (p = 0.034), parenchymal impairment visually established by the observer (p = 0.029), and the chest CT severity score (p = 0.029). Between subgroups with respiratory failure, a significant difference was found in the level of D-dimers (p = 0.019, predominant in the first wave) and the number of diabetics (p = 0.024, predominant in the second wave). The results of the univariate analysis between the first and second waves are presented in Table 2.

**Table 2:** Univariate analysis between the two waves. SD: standard deviation; ICU: intensive care unit; COPD: chronic obstructive pulmonary disease; HBP: high blood pressure. AI: artificial intelligence; CT: computed tomography

Variables	First wave		Second wave		P-value
	n (%)	Average (SD)	n (%)	Average (SD)	
Female	50 (49.5)		51 (50.5)		0.89
Male	51 (50.5)		50 (49.5)		
Age	101	65	101	67	>0.05
Admission in COVID unit	23 (23)		22 (22)		0.84
Admission in ICU	13 (13)		20 (21)		0.14
Mortality	22 (23)		26 (27)		0.51
Complications	47 (49)		43 (51)		0.77
Asthma	3 (3)		13 (13)		0.009
Renal failure	30 (30)		23 (23)		0.28
COPD	11 (11)		8 (8)		0.47
Heart	40 (40)		29 (29)		0.10
Obesity	28 (28)		42 (42)		0.038
Diabetes	32 (32)		35 (35)		0.65
HBP	50 (50)		64 (64)		0.046
Neoplastic atcd	12 (12)		6 (6)		0.14
Active neoplasia	2 (2)		8 (8)		0.054
Ground-glass opacity	99 (98)		100 (99)		0.56
Crazy paving	60 (59)		71 (70)		0.10
Consolidation	55 (54)		53 (52)		0.78
Spider web sign	30 (30)		32 (32)		0.76
Adenopathy	16 (16)		24 (24)		0.16
Pleural effusion	10 (10)		8 (8)		0.62
Visual estimation (%)		26(19)		34.5 (22)	>0.05
AI estimation (%)		19.7(20)		23.3 (21)	>0.05
Chest CT severity score		10(5)		10.8 (5)	>0.05

In the first wave, the risk factors established by logistic regression for mortality included respiratory failure (odds ratio (OR) = 6.7, p = 0.003) and chest CT severity score (OR = 1.2, p = 0.003). For respiratory failure, only chest CT severity score was considered a risk factor (OR = 1.25, p = 0.0001). In the second wave, for mortality, risk factors included respiratory failure (OR = 5.9, p = 0.022) and complications (OR = 20.8, p = 0.006). For respiratory failure, only diabetes (OR = 12.7, p = 0.002) was a risk factor. Lung damage was characterized by 98% and 99% ground-glass opacities per wave, respectively, with an average involvement of four lobes. Mixed distribution predominated in the second wave (p = 0.017). The frequency of the other scanning elements is shown in Table 2. The mean values of the different biological parameters in our cohort were hemoglobin 12.9 g/dL (SD = 2.2), C-reactive protein 89.5 mg/dL (SD 78.4), mild lymphopenia 1,093 (SD = 654), normal neutrophil and platelet counts, increased D-dimer 1,752 ng/mL (SD = 3,416), slightly decreased saturation to 91% (SD = 7), and average oxygen requirement of 2.4 L (SD = 3.6).

**Figure 3:**
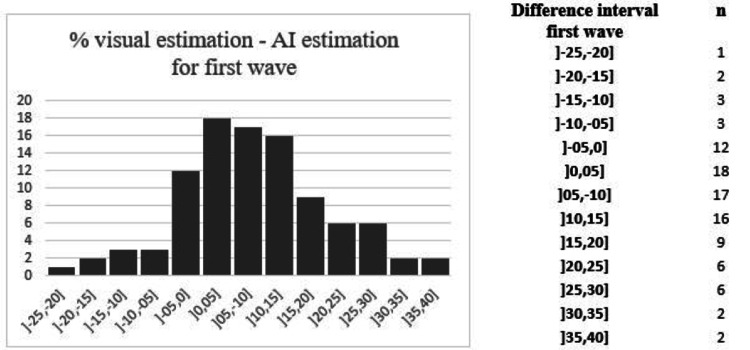
Difference between visual estimation and AI during the first wave. AI: artificial intelligence

**Figure 4:**
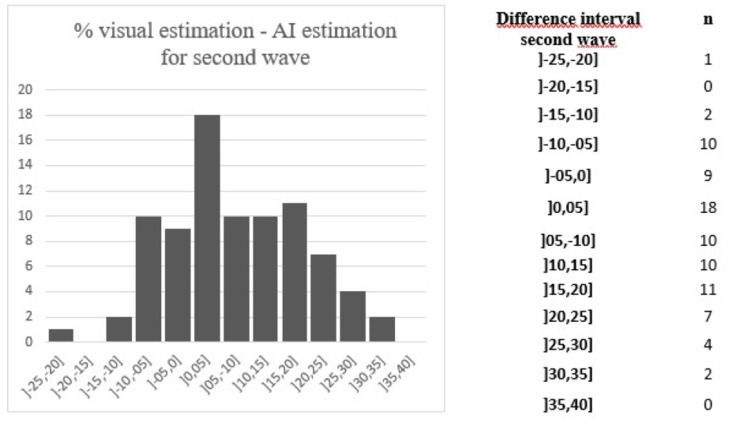
Difference between visual estimation and AI during the second wave. AI: artificial intelligence

The most frequent complications included respiratory failure (17/101 vs. 14/101), cardiac decompensation (11/101 vs. 8/101), and bacterial infections (8/101 vs. 13/101). Among the least frequent were Kawasaki-like manifestations (one in the first wave), encephalopathies (two in the first wave), pulmonary embolism (two vs. two), and pericarditis (one in the second wave). Figures 3, 4 show the estimated differences in the percentage of pulmonary damage quantization between the observers and AI (negative values correspond to underestimates and positive values to overestimates). An error interval greater than 10% was found in 40% of the cases during both waves. The maximum overestimate and underestimate values were 39.2% and -23% for the first wave and 33.48% and -25.08% for the second wave, respectively. For 8% of the patients in our cohort (14/181), a discrepancy between the observer and AI implied a false-positive hospitalization criterion. For 21 patients, parenchymal disease quantization did not appear on the examination report.

## Discussion

Ground-glass hyperdensities represent an average of the CT system for hyperdensities smaller than the spatial resolution of the system. They may originate from the alveolar, interstitial, or capillary compartment, which explains their low specificity [11]. In our cohort, there was a significant difference in the distribution of lesions between the two waves (mixed distribution predominating in the second wave). The presence of central ground-glass opacities can be explained by bronchial and vascular syndrome, active heart problems and cardiac complications were not different, and coinfection by other respiratory viruses reported in the literature. Davis et al. [12] have shown a variable coinfection frequency (16.8-26.8%), which can explain this difference. The second wave occurred in autumn when multiple respiratory viruses are endemic in our region.

The following three stages of radiological evolution have been described: the first, called the rapid progressive period, lasting from one to seven days after the onset of symptoms; the second, called the advanced period (8-14 days), where the pulmonary damage is more severe; and the third after the 14th day when pulmonary damage begins to decrease [13].

In this study, we investigated the first and second wave clinical and radiological stages, the risk factors, including the presence of respiratory failure, complications, diabetes, and extent of radiological involvement, and the chest CT severity score. Biology during the initial period remains slightly inflammatory, characterized by a higher-than-normal level of D-dimers and moderate lymphopenia. This biological presentation was constant during both waves, highlighting the unpredictability of the disease, which evolved rapidly in the second phase without any biological marker able to be the initial predictor of its decline. Risk factors for poor prognosis include age, male sex, heart disease, chronic pneumonia, the presence of two or more comorbidities, high Sequential Organ Failure Assessment score, and obesity [14-16]. In our cohort, we found a likelihood between the two waves despite a significant difference in some comorbidities associated with a poor prognosis (asthma and obesity in the second wave).

The criteria for hospitalization are multiple and are primarily clinical-biological [17,18], although a pejorative impairment of imaging remains a criterion used in some institutions [19]. At CHU Brugmann, a parenchymal threshold of 50% is used as a criterion for hospitalization. AI can be used at different levels of COVID-19 control in radiology [20]. The most common applications include lesion detection, quantitative estimation, and differential diagnosis with other lung pathologies [21].

In our cohort, the interobserver (AI and observers) match was low, with an error greater than 10% in approximately 40% of cases. Moreover, in 8% of cases, this discrepancy resulted in a false-positive hospitalization criterion.

Given the utility of the chest CT severity score as a predictor for respiratory failure and mortality [8], AI can be a useful tool for the estimation of pulmonary involvement and calculation of the chest CT severity score.

Wawina-Bokalanga et al. analyzed the genome of SARS-CoV-2 and its evolution during the first wave in Belgium, identifying more than 42 different SARS-CoV-2 lineages [22]. However, the first variants, alpha and beta, with likely significant impact in Europe according to the European Center for Disease Prevention and Control in September 2020 [23] and according to the report of January 21, 2021, of the Belgium genomic monitoring group of SARS-CoV-2, the variants of concern represented 10-15% of the cases [24].

Our study did not show significant changes in patient status during the initial period of the disease, either radiologically, clinically, or epidemiologically, or statistical differences in outcome (mortality, respiratory failure, and complications).

A limitation of the study is its sample size. This is a retrospective monocentric study, and we are not aware of the genotype of SARS-CoV-2 that infected patients because it was not sequenced at the time in our institution.

## Conclusions

In our cohort, between March and December 2020, for the majority of the parameters analyzed, we did not record significant changes between the two waves, either radiologically or clinico-biologically. This trend suggests that the mutations in progress may not be more virulent, at least in the time window explored. AI in daily practice is a useful tool for estimating pulmonary damage from COVID-19 pneumonia and can be one of the hospitalization criteria in an environment where hospital beds are limited.
